# Age-dependent regional retinal nerve fibre changes in *SIX1/SIX6* polymorphism

**DOI:** 10.1038/s41598-020-69524-8

**Published:** 2020-07-27

**Authors:** Jason Charng, Mark Simcoe, Paul G. Sanfilippo, R. Rand Allingham, Alex W. Hewitt, Chris J. Hammond, David A. Mackey, Seyhan Yazar

**Affiliations:** 1 Centre for Ophthalmology and Visual Science (incorporating Lions Eye Institute), The University of Western Australia, 2 Verdun St, Perth, WA 6009 Australia; 2 Department of Twin Research and Genetic Epidemiology, Kings College London, London, UK; 3 Centre for Eye Research Australia, Royal Victorian Eye and Ear Hospital, University of Melbourne, Melbourne, 3002 Australia; 4 Department of Ophthalmology, Duke University Medical Center, Durham, NC USA; 5 School of Medicine, Menzies Research Institute Tasmania, University of Tasmania, Hobart, Australia; 6 Garvan Institute of Medical Research, Sydney, Australia

**Keywords:** Epidemiology, Genetics research

## Abstract

*SIX1/SIX6* polymorphism has been shown to be associated with glaucoma. Studies have also found that, in older adults, retinal nerve fibre layer (RNFL) thickness is significantly thinned with each copy of the risk allele in *SIX1/SIX6*. However, it is not known whether these genetic variants exert their effects in younger individuals. Comparing a healthy young adult with an older adult cohort (mean age 20 vs 63 years), both of Northern European descent, we found that there was no significant RNFL thinning in each copy of the risk alleles in *SIX1/SIX6* in the eyes of younger individuals. The older cohort showed an unexpectedly thicker RNFL in the nasal sector with each copy of the risk allele for both the *SIX1* (rs10483727) and *SIX6* (rs33912345) variants. In the temporal sector, thinner RNFL was found with each copy of the risk allele in rs33912345 with a decrease trend observed in rs10483727. Our results suggest that *SIX1/SIX6* gene variants exert their influence later in adult life.

## Introduction

Genome-wide association (GWA) studies have identified multiple genetic variants that increase susceptibility to Primary Open-Angle Glaucoma (POAG)^1–3^. Among these, the rs10483727 variant in the *SIX1* region was significantly associated with POAG diagnosis and progression in a quantitative trait GWA study^4^ and with risk of POAG in a later case-control GWA study^3^. More importantly, the rs10483727 variant was associated with POAG across different ethnicities^2,5–8^. However, to date, no causal variants driving its association with POAG have been identified. Similarly, the effect of rs10483727 on protein function remains unknown. The missense variant rs33912345 (Asn141His), found in the *SIX6* locus^9^, is in strong linkage disequilibrium with rs10483727. It is also associated with POAG and has been shown to alter the function of the SIX6 protein^10^. *SIX6* is involved in the differentiation and survival of retinal ganglion cells^11,12^, which are the primary cells affected in POAG. Both *SIX1* and *SIX6* belong to the family of sine oculis (SIX) transcription factors with six members in mammals (*SIX1 to SIX6*). All members of the family are characterized by the presence of two highly conserved domains: a homeobox domain and a *SIX* domain^13,14^. *SIX1* is expressed in several tissues, including the optic vesicles and the limb mesenchyme, but it is poorly expressed in the eye. In contrast, studies in different species, including humans, have shown that *SIX6* is highly expressed in the developing retina, especially in the retinal ganglion and the amacrine cells^11,15,16^. Genetic inactivation of *SIX6* has been shown to result in hypoplasia of the mice retina, which subsequently lead to absence of the optic nerves^16^.

It is well established that POAG results in thinning of the retinal nerve fibre layer (RNFL)^17,18^, thus RNFL assessment has become indispensable in POAG management^19^. Polymorphisms in *SIX6* and *SIX1* have been reported to result in significant RNFL thinning but the region of RNFL thinning has varied depending on the study. More specifically, each copy of the risk allele was associated with significant thinning of global peripapillary RNFL for rs33912345 in large European^20^ and Singaporean^21^ cohorts but not in a Japanese population^22^. In addition, the above studies also partitioned global RNFL into inferior, nasal, superior and temporal quadrants and found significant thinning with each copy of the rs33912345 risk allele and the superior as well as the inferior quadrants in the Singaporean study^21^ but significant thinning was found in the temporal and inferior quadrants in the Japanese study^22^. In rs10483727, an European cohort study reported significant global RNFL thinning with each copy of the risk allele as well as the inferior and superior sectors^23^ but the study cohort contained a large portion of glaucoma cases and suspects (~ 80%).

To date, the association between RNFL thickness and *SIX1/SIX6* variants have only been reported in older individuals (mean age > 50 years)^20–23^. Hence it is not known whether these genetic variants exert their effects in younger individuals. In this study, we investigate the effect of age on *SIX1/SIX6* polymorphism by examining the RNFL thickness in two large adult cohorts of similar descent but with substantial age difference.

## Results

A total of 2,541 individuals; (943 from younger cohort, the Raine Study Gen2 (RS GEN2) and 1598 from the older cohort, TwinsUK adult twin Registry (TwinsUK)) for whom both RNFL and genotypic data were available were included in the analysis. Demographic, genotypic and ocular characteristics are presented in Table 1. In the younger RS GEN2 cohort, the mean age was 20.0 years (range: 18.3–22.1) and 488 participants (52%) were female. In the older TwinsUK cohort, the average age was 62.6 years (range: 21.7–89.7) and the proportion of female participants (91%) was much greater than in the RS GEN2 (52%) cohort. While there were no glaucoma suspects or cases in the RS GEN2, the prevalence of self-report glaucoma from questionnaire data in TwinsUK was 1.5%.

**Table 1:** Characteristics of study populations.

		RS GEN2 n = 943	TwinsUK n = 1598
Demographic characteristics	Age in years (range)	20.0 (18.3–22.1)	62.6 (21.7–89.7)
	Sex (female)	488 (52%)	1,455 (91%)
Genotypic characteristics	**rs33912345**		
	Genotype A/A	338 (35.8%)	545 (34.1%)
	Genotype A/C	466 (49.4%)	774 (48.4%)
	Genotype C/C	139 (14.7%)	279 (17.5%)
	**rs10483727**		
	Genotype C/C	334 (35.4%)	548 (34.3%)
	Genotype C/T	478 (50.7%)	772 (48.3%)
	Genotype T/T	131 (13.9%)	278 (17.4%)
Ocular characteristics	**Peripapillary RNFL thickness (μm)**		
	Global	100.4 ± 9.8	95.5 ± 9.9
	Inferior sector	126.7 ± 16.9	119.0 ± 14.9
	Superior sector	124.3 ± 15.8	115.3 ± 14.4
	Nasal sector	79.3 ± 13.8	75.4 ± 13.1
	Temporal sector	71.3 ± 10.4	72.1 ± 9.4
	**Central corneal thickness (μm)**	539.0 ± 32.5	539.6 ± 35.7
	**Intraocular pressure (mmHg)**	15.5 ± 3.2	13.4 ± 3.1
	**Spherical equivalent (D)**	0.00 ± 1.44	0.22 ± 2.6

The *SIX6* variant rs33912345 had C allele frequency of 39.4% in the RS GEN2 cohort. It was in strong linkage disequilibrium with the previously identified variant rs10483727 (R^2^ = 0.98). The genotype frequencies of *SIX6* rs33912345 among the 943 participants were 338 (35.8%) for genotype A/A, 466 (49.4%) for A/C, and 139 (14.7%) for C/C. A similar distribution of genotype frequencies was observed in the TwinsUK older cohort (Table 1). In RS GEN2, for rs10483727, the genotype frequency for C/C, C/T and T/T were 334 (35.4%), 478 (50.7%) and 131 (13.9%), respectively. The genotype distribution for both rs10483727 and rs33912345 in the TwinsUK cohort were similar to that of the RS GEN2 cohort.

In RS GEN2, in both global and sectorial RNFL thicknesses, there was no difference between the three genotypes for both rs33912345 and rs10483727 (Table 2), as confirmed by one-way ANOVA (*p* value range 0.24–0.90). TwinsUK data also showed that there was no significant difference in global and sectorial RNFL thicknesses in both rs33912345 and rs10483727 between the three genotypes (*p* value range 0.06–0.98). Unexpectedly, the nasal sector in both rs33912345 and rs10483727 was thinner with both copies of the major allele, although no significance was detected (rs33912345: A/A 74.8 ± 12.5 µm, A/C 76.1 ± 13.1 µm, C/C 76.9 ± 13.8 µm, *p* = 0.06; rs10483727: C/C 74.9 ± 12.4 µm, C/T 75.9 ± 13.2 µm, T/T 77.1 ± 13.7 µm, *p* = 0.07). Individuals with the risk allele genotype of rs33912345 showed a trend of thinner RNFL in the temporal sector, again not statistically significant (A/A 72.8 ± 10.0 µm, A/C 72.0 ± 9.0 µm, C/C 71.2 ± 9.8 µm, *p* = 0.06).

**Table 2:** Peripapillary RNFL thickness by *SIX1/SIX6* region variant genotypes.

	Global	Inferior sector	Superior sector	Nasal sector	Temporal sector
**rs33912345** **RS GEN2**					
A/A (n = 338)	100.9 ± 10.1	127.3 ± 17.7	125.3 ± 16.0	79.4 ± 14.0	71.5 ± 10.0
A/C (n = 466)	100.3 ± 9.6	126.5 ± 16.3	123.5 ± 15.5	79.7 ± 13.8	71.2 ± 10.8
C/C (n = 139)	99.9 ± 10.0	125.7 ± 17.0	124.8 ± 16.3	77.6 ± 13.7	71.3 ± 10.1
*p* value*	0.560	0.611	0.258	0.279	0.898
**TwinsUK**					
A/A (n = 545)	95.5 ± 9.8	118.8 ± 15.2	115.7 ± 14.7	74.8 ± 12.5	72.8 ± 10.0
A/C (n = 774)	95.8 ± 9.6	119.5 ± 14.9	115.6 ± 13.9	76.1 ± 13.1	72.0 ± 9.0
C/C (n = 279)	95.5 ± 10.5	119.0 ± 15.2	114.9 ± 14.2	76.9 ± 13.8	71.2 ± 9.8
*p* value*	0.830	0.692	0.726	0.061	0.063
**rs10483727** **RS GEN2**					
C/C (n = 334)	101.0 ± 10.1	127.4 ± 17.7	125.4 ± 15.9	79.4 ± 13.8	71.6 ± 10.1
C/T (n = 478)	100.3 ± 9.7	126.4 ± 16.3	123.5 ± 15.5	79.7 ± 13.9	71.1 ± 10.7
T/T (n = 131)	99.9 ± 10.0	125.8 ± 17.2	124.6 ± 16.6	77.4 ± 13.4	71.4 ± 10.0
*p* value*	0.460	0.556	0.242	0.243	0.798
**TwinsUK**					
C/C (n = 548)	95.6 ± 9.8	118.9 ± 15.1	115.8 ± 14.5	74.9 ± 12.4	72.7 ± 10.0
C/T (n = 772)	95.7 ± 9.7	119.4 ± 15.0	115.4 ± 13.9	75.9 ± 13.2	72.0 ± 9.0
T/T (n = 278)	95.6 ± 10.5	119.1 ± 15.2	115.0 ± 14.3	77.1 ± 13.7	71.3 ± 9.9
*p* value*	0.980	0.835	0.733	0.067	0.121

*One-way ANOVA of RNFL thickness between the three genotypes.

Linear regression showed that, in rs33912345, the younger RS GEN2 cohort showed non-significant decrease in global and all four RNFL sectors with each copy of the C risk allele (Table 3). However, the temporal (β =  − 0.73, *p* = 0.04) quadrant was significantly thinner with the C risk allele in the older TwinsUK cohort. In agreement with the genotype data presented in Table 2, each copy of the risk allele was associated with increase in RNFL thickness (β =  + 1.02, *p* = 0.03). Similarly, the younger cohort showed no significant thinning with each copy of the T risk allele in rs10483727 (Table 4). In the older cohort, the nasal sector showed significant thicker RNFL with the risk allele (β =  + 0.99, *p* = 0.04). A RNFL thinning trend, close to statistical significance, was observed in the temporal sector (β =  − 0.67, *p* = 0.06).

**Table 3:** Association of rs33912345 with peripapillary retinal nerve fibre layer thickness.

	β^a^	SE	95% CI	*p* value
**RS GEN2**^b^				
	− 0.33	0.47	− 1.24, 0.59	0.482
Superior	− 0.37	0.75	− 1.85, 1.11	0.623
Inferior	− 0.55	0.80	− 2.12, 1.02	0.491
Temporal	− 0.06	0.50	− 1.04, 0.93	0.913
Nasal	− 0.47	0.66	− 1.76, 0.82	0.474
**TwinsUK**^c^				
Global	− 0.05	0.35	− 0.74, 0.64	0.889
Superior	− 0.40	0.51	− 1.40, 0.60	0.436
Inferior	− 0.06	0.55	− 1.14, 1.02	0.921
Temporal	− 0.73	0.36	− 1.44, − 0.02	0.041
Nasal	+ 1.02	0.47	+ 1.94, + 0.10	0.031

^a^β, changes in RNFL thickness (in µm) per copy of the risk allele.

^b^Model 1: RNFL = α + age + sex + spherical equivalent + principal components + rs33912345_C.

^c^Model 2: RNFL = α + age + sex + spherical equivalent + population structure + relatedness + rs33912345_C.

**Table 4:** Association of rs10483727 with peripapillary retinal nerve fibre layer thickness.

	β^a^	SE	95% CI	*p* value
**RS GEN2**^b^				
Global	− 0.43	0.47	− 1.36, 0.50	0.364
Superior	− 0.56	0.76	− 2.06, 0.94	0.464
Inferior	− 0.65	0.81	− 2.24, 0.94	0.423
Temporal	− 0.14	0.51	− 1.14, 0.86	0.786
Nasal	− 0.51	0.67	− 1.82, 0.80	0.444
**TwinsUK**^c^				
Global	− 0.06	0.35	− 0.75, 0.63	0.873
Superior	− 0.47	0.51	− 1.47, 0.53	0.355
Inferior	− 0.03	0.55	− 1.11, 1.05	0.958
Temporal	− 0.67	0.35	− 1.36, 0.02	0.057
Nasal	+ 0.99	0.47	+ 1.91, + 0.07	0.035

^a^β, changes in RNFL thickness (in µm) per copy of the risk allele.

^b^Model 1: RNFL = α + age + sex + spherical equivalent + principal components + rs10483727_T.

^c^Model 2: RNFL = α + age + sex + spherical equivalent + population structure + relatedness + rs10483727_T.

## Discussion

Large GWA studies allow the identification of genetic variants that are associated with common, complex diseases of old age. However, in the case of POAG and *SIX1/SIX6*, it is not known when these genetic variants start exerting their effects^24^. Studies have reported that individuals with risk allele in *SIX1/SIX6* are more susceptible to glaucoma^1,3,25^ and have thinner RNFL^20–23^. Note that these RNFL studies were conducted in older individuals, hence it is not clear whether thinner RNFL found in the older individuals with *SIX1/SIX6* variants is due to these individuals being born with thinner RNFLs or due to a faster rate of RNFL degeneration as they age. Our data suggests the latter scenario, as there was no significant decrease in RNFL thickness for both rs33912345 and rs10483727 variants in the younger cohort but we found evidence of sectorial RNFL thinning in rs33912345 in the older cohort, with a trend of sectorial thinning in rs10483727. This suggests that the effect *SIX1/SIX6* variant has on RNFL thickness is age-dependent and does not manifest until later in adult life.

We found, in our older cohort, each copy of the rs10483727 risk allele was associated with significantly thicker nasal RNFL. Temporal RNFL was trending thinner, albeit not significantly, with each copy of the risk allele. This finding differs to a previous study in individuals of European descent (n = 231, mean age 65.1 years), which reported that each copy of the rs10483727 risk allele was associated with significant thinning of the inferior and superior but not nasal or temporal sectors^23^. The study cohort was relatively small and skewed towards subjects with glaucoma (46 healthy individuals, 101 glaucoma suspects, 84 clinically diagnosed glaucoma), which differs to the current study. The authors did not report the proportion of T-risk in their cohort. However, given the high proportion of glaucoma cases (suspect and confirmed) and that these glaucoma cases present with a higher proportion of T risk allele, it can be assumed that the study population contains higher T risk allele proportion than our TwinsUK cohort. In addition, the RNFL thickness in the aforementioned study is thinner than our cohort for all three genotypes, most likely also due to the higher proportion of glaucoma cases. Hence these factors could combine to result in different findings between the two studies. Nevertheless, this does not explain our atypical finding of thicker nasal RNFL with each copy of the rs10483727 risk allele in the TwinsUK cohort, which we have no satisfactory explanation for but it may be partly attributed to the higher variability in nasal RNFL measurement^26^. In rs33912345, we found significantly thinner RNFL in the temporal but thicker RNFL in the nasal sector with each copy of the risk allele in the older TwinsUK cohort, which again differs from previous reports. In a Japanese population^22^ (n = 2,306, mean age 57.6 years), significant thinning was found between each copy of the rs33912345 risk allele in both inferior and temporal RNFL sectors. In contrast, a Singaporean-based study^21^ (n = 1,243, mean age 55.0 years) showed significant thinning with rs33912345 risk allele in both superior and inferior sectors. One possible explanation of the difference in sectoral thinning between the different studies could be the difference in allele distribution. In support of our postulation, the rs33912345 genotype distribution of the Singaporean study (A/A 4.0%, A/C 32.1%, C/C 63.9%) is vastly different when compared to the TwinsUK cohort (A/A 34.1%, A/C 48.4%, C/C 17.5%). The higher prevalence of the C risk allele in the Singaporean population may explain why global RNFL thinning was observed but not in the TwinsUK cohort. Allele distribution was not provided in the Japanese study. Further investigation is required to confirm whether difference in genotype distribution can result in difference in sectorial RNFL thinning.

A key strength of this study is the inclusion of two large adult cohorts with similar ancestry but with a clear age gap between the two groups. The extensive ocular and genetic data collected in both groups allowed us to perform similar analyses, which enabled direct comparison between the two populations. Furthermore, normative RNFL thickness values are reported in two large populations of different ages, providing reference value for future studies. The main limitation of this study is the cross-sectional design. A longitudinal study will allow us to define the age range when RNFL thickness in adults with risk alleles deviates from those with major alleles, which could shed light on glaucoma disease mechanism and advise on age for glaucoma screening.

In contrast to evidence from the older populations, we found no effects of the *SIX1/SIX6* gene variants rs33912345 and rs10483727 on peripapillary RNFL thickness of young adults. However, we found evidence for sectorial thinning in the older cohort with each copy of the risk allele in an older cohort with similar ancestry, albeit with different RNFL profile compared to previous studies in other ethnicities. Our study suggests that *SIX1/SIX6* gene variants exert their influence later in adult life. Further research should concentrate on validating these results as well as identifying functional pathways involving *SIX1/SIX6* gene and other common POAG risk factors.

## Methods

### The Raine Study Gen2

The Raine Study is a multigenerational, longitudinal study conducted in Perth, Western Australia^27^. At the 20-year follow-up of the cohort, 1,344 participants were enrolled in RS GEN2, a cross-sectional study of eye diseases in young adults^28^. All participants underwent a comprehensive standardised ocular examination. This included optical coherence tomography (OCT) measurements of the peripapillary RNFL by Spectralis (Heidelberg Engineering GmbH, Heidelberg, Germany). In each eye, a circular scan (diameter 3.5 mm) centred on the optic nerve was acquired by averaging 16 consecutive frames in real time. RNFL thickness was determined in four quadrants (superior, inferior, temporal, nasal) around the optic disc automatically using a Heidelberg Eye Explorer (HEYEX) software version 6.7.12.0 as well as global mean RNFL thickness. Intraocular pressure (IOP) with an ICare TAO1i Tonometer (Icare Finland, Oy, Helsinki, Finland), central corneal thickness (CCT) with an Oculus Pentacam (Optikgerate GmbH, Wetzlar, Germany) and axial length using noncontact partial coherence interferometry (IOL Master V.5; Carl Zeiss Meditec AG, Jena, Germany) were also acquired at the same visit.

Genotyping was performed on 1593 participants, including those who did not attend the vision assessments, using the Illumina Human 660W-quad BeadChip (Illumina, Inc., San Diego, CA, USA), which included the *SIX6* missense variant rs39912345 and *SIX1/SIX6* variant rs10483727. After standard quality control, the cleaned genotypic datasets included 1,494 individuals. Details of the quality control step have been described previously^29^.

Only subjects with both RNFL and genotyping data as well as of Northern European descent are included in the analysis.

### TwinsUK cohort study

The TwinsUK Adult Twin Registry is based at St Thomas’ Hospital, London. Participants were unaware of any hypotheses or proposals for specific studies; only later were they invited to have an eye examination. The St Thomas’ Hospital Local Research Ethics Committee approved the study, and all the twin participants volunteered to join the TwinsUK Registry and gave informed consent to attend the hospital for phenotyping and for their data to be used for scientific research. Spherical equivalent was calculated from autorefraction data (ARM-10, Tagaki Ltd, Takaoka, Japan). RNFL was assessed with the OCT (Optovue iVue sd-OCT, Fremont, CA, USA) using a circular scan (4.9 mm diameter) centred at the optic nerve head; both global and quadrant thicknesses were determined by the Optovue algorithm^30^.

Genotyping of the TwinsUK cohort was done with a combination of Illumina HumanHap300 and HumanHap610Q chips. Intensity data for each of the arrays were pooled separately and genotypes were called with the Illuminus32 calling algorithm, thresholding on a maximum posterior probability of 0.95. Imputation was performed using the IMPUTE 2.0^31^ software package using haplotype information from the Haplotype Reference Consortium panel, pre-phased using ShapeIT^32^. Analyses were done using GEMMA^33^, which implements a mixed model that adjusts for similarity between individuals.

Only subjects with both RNFL and genotyping data as well as of Northern European descent are included in the analysis.

### Statistical analysis

Average of right and left eye values were included in the analysis for both studies, regardless of glaucoma status. In both studies, potential covariates affecting both the global- and sectorial-specific RNFL measures were evaluated using scatterplots and a correlation matrix (data not shown). An additive genetic model was constructed to determine the association between rs33912345 and RNFL thickness as a quantitative trait whereby the number of minor allele copies was coded into 0, 1 and 2 (the C allele of rs39912345) and used in a linear regression model adjusted for age, sex and significant predictors of RNFL measures. In the case of the TwinsUK cohort, relatedness was also taken into account. RNFL measurements were compared between genotype groups using a trend test. Logistic regression analyses were also performed with case/control status as an outcome (dependent variable) and rs33912345 genotype as the predictor variable. The same additive genetic modelling was performed for rs10483727 in the RS GEN2 and TwinsUK data.

The statistical analyses were performed using the statistical software R version 3.6.2 (R Foundation for Statistical Computing, https://www.r-project.org/ [in the public domain]) in the RS GEN2 and STATA14 statistical package (www.stata.com) in TwinsUK. A *p* value < 0.05 was considered statistically significant.

### Ethics statement

This study utilised data from the Raine Study Gen2 (RS GEN2) and the TwinsUK adult twin Registry (TwinsUK). Both studies were conducted in accordance with the tenets of the Declaration of Helsinki. Individual study protocols were approved by the University of Western Australia Human Research Ethics Committee and the Guys and St. Thomas' ethics committee for the RS GEN2 and TwinsUK, respectively. Informed consent was obtained from all participants prior to the ocular examination session.
